# An individually tailored family-centered intervention for pediatric obesity in primary care: study protocol of a randomized type II hybrid effectiveness–implementation trial (Raising Healthy Children study)

**DOI:** 10.1186/s13012-017-0697-2

**Published:** 2018-01-15

**Authors:** Justin D. Smith, Cady Berkel, Neil Jordan, David C. Atkins, Shrikanth S. Narayanan, Carlos Gallo, Kevin J. Grimm, Thomas J. Dishion, Anne M. Mauricio, Jenna Rudo-Stern, Mariah K. Meachum, Emily Winslow, Meg M. Bruening

**Affiliations:** 1 Department of Psychiatry and Behavioral Sciences, Northwestern University Feinberg School of Medicine, Chicago, IL USA; 2 Department of Preventive Medicine, Northwestern University Feinberg School of Medicine, Chicago, IL USA; 3 Department of Pediatrics, Northwestern University Feinberg School of Medicine, Chicago, IL USA; 4 REACH Institute, Department of Psychology, Arizona State University, Tempe, AZ USA; 5 Department of Psychiatry and Behavioral Sciences, University of Washington School of Medicine, Seattle, WA USA; 6 Department of Electrical Engineering and Computer Science, University of Southern California, CA, Los Angeles USA; 7 Department of Nutrition, Arizona State University, Tempe, AZ USA

**Keywords:** Family Check-Up 4 Health, Primary care, Hybrid effectiveness–implementation trial, Pediatric obesity, Integrated care, Coordinated care

## Abstract

**Background:**

Pediatric obesity is a multi-faceted public health concern that can lead to cardiovascular diseases, cancers, and early mortality. Small changes in diet, physical activity, or BMI can significantly reduce the possibility of developing cardiometabolic risk factors. Family-based behavioral interventions are an underutilized, evidence-based approach that have been found to significantly prevent excess weight gain and obesity in children and adolescents. Poor program availability, low participation rates, and non-adherence are noted barriers to positive outcomes. Effective interventions for pediatric obesity in primary care are hampered by low family functioning, motivation, and adherence to recommendations.

**Methods:**

This (type II) hybrid effectiveness–implementation randomized trial tests the Family Check-Up 4 Health (FCU4Health) program, which was designed to target health behavior change in children by improving family management practices and parenting skills, with the goal of preventing obesity and excess weight gain. The FCU4Health is assessment driven to tailor services and increase parent motivation. A sample of 350 families with children aged 6 to 12 years who are identified as overweight or obese (BMI ≥ 85th percentile for age and gender) will be enrolled at three primary care clinics [two Federally Qualified Healthcare Centers (FQHCs) and a children’s hospital]. All clinics serve predominantly Medicaid patients and a large ethnic minority population, including Latinos, African Americans, and American Indians who face disparities in obesity, cardiometabolic risk, and access to care. The FCU4Health will be coordinated with usual care, using two different delivery strategies: an embedded approach for the two FQHCs and a referral model for the hospital-based clinic. To assess program effectiveness (BMI, body composition, child health behaviors, parenting, and utilization of support services) and implementation outcomes (such outcomes as acceptability, adoption, feasibility, appropriateness, fidelity, and cost), we use a multi-method and multi-informant assessment strategy including electronic health record data, behavioral observation, questionnaires, interviews, and cost capture methods.

**Discussion:**

This study has the potential to prevent excess weight gain, obesity, and health disparities in children by establishing the effectiveness of the FCU4Health and collecting information critical for healthcare decision makers to support sustainable implementation of family-based programs in primary care.

**Trial registration:**

NCT03013309 ClinicalTrials.gov

**Electronic supplementary material:**

The online version of this article (10.1186/s13012-017-0697-2) contains supplementary material, which is available to authorized users.

## Background

Childhood obesity has become the leading preventable cause of death worldwide [1]. Poor diet/nutrition and physical inactivity are the leading and preventable contributors to obesity and are among the principal causes of chronic disease and mortality for youths [2]. Obesity is also related to psychosocial issues in youth, such as depression and academic disengagement [3–5]. In the USA in 2013–2014, it was estimated that 18% of all 6–11-year-old children were obese and that a disproportionate number are disadvantaged economically and socially [6]. Obesity and its health consequences are disproportionately distributed across the USA; Mexican Americans, American Indians, and African Americans have the highest prevalence rates of obesity in childhood at 41.1 to 56.3% [6–9]. Social determinants of health [10] play a key role in the childhood obesity and contribute substantially to disparities. In the social environment, stress and discrimination are acutely experienced by minority and low-income communities in the USA and are linked with childhood obesity [11]. Physical environment barriers include limited access to safe areas for play and fresh, nutritious foods [12]. Finally, access to health services that can prevent obesity and its sequelae [13] is limited for members of underserved groups [14].

While addressing the social determinants of health is an ongoing task, significant evidence supports the effectiveness of lifestyle modification on cardiometabolic risk factors, with even small changes yielding significant impact [15]. Recently published reviews [16, 17] indicate that youth health behaviors and obesity may be improved when parents attend and are directly involved with services [18] and are provided training in the skills required to support lifestyle modification in accordance with expert guidelines [16, 19–21].

Primary care is a promising context for improving parenting behaviors linked to child health [22–24]. Most children access a primary care provider annually [25, 26], caregivers are present at visits, and physicians have a high level of perceived authority among parents who look to them for advice on children’s health. The National Academy of Medicine, the American Academy of Pediatrics, and the Endocrine Society, among others, promote a family-centered intervention in primary care [27–29]. Despite the appeal of a family-centered approach [30, 31], several barriers hinder implementation of such programs in real-world healthcare systems [29, 32]. For example, healthcare providers report inadequate time, training, and resources to effectively work with parents to address childhood obesity [33]. Few comprehensive and prospective evaluations of the implementation of weight management programs in primary care have been done, despite such factors as inadequate cost evaluation being a top reason for failure to adopt behavioral interventions [34] and the general concern about delivery with sufficient fidelity to preserve effectiveness [35]. To have a population impact on pediatric obesity, there is a pressing need for scalable programs that can be delivered in primary care and engage parents in behavior change strategies for children [32].

### The Family Check-Up for Health program

The Family Check-Up 4 Health (FCU4Health) is an adaptation and enhancement of the original Family Check-Up (FCU), an evidence-based, family-centered intervention with empirical evidence of effectiveness and feasible delivery in real-world systems that provide care for culturally diverse, low-income families [36–39]. Initially designed and tested as a behavioral health intervention, the FCU employs motivational interviewing (MI) to engage parents in family management strategies and community-based support services [40]. Extensive data document the FCU’s clinical effectiveness and high rates of engagement among underserved and ethnic minority families [36–38, 41–43]. Although not targeted by the program, the FCU demonstrated long-term collateral effects on nutrition (ages 2–5) and obesity (ages 2 to 10 years) [44] and on those from adolescence to early adulthood (ages 12 to 22 years) [13, 44]. Improvement in parent–child relationships and positive behavior support mediated the effects of the FCU on nutrition and obesity in both developmental periods. Based on these findings, the FCU4Health was developed to specifically target pediatric obesity in primary care, the front-line service setting for prevention of obesity and excess weight gain [29]. This involved enhancing the content to focus on helping parents support child and family behaviors that lead to a healthy weight, and adapting the delivery strategy for the primary care context (Smith JD, Berkel C, Rudo-Stern J, Montaño Z, Mauricio AM, Dishion TJ, St. George SM, Prado G, Chiapa A, Bruening MM, The Family Check-Up 4 Health (FCU4Health): The process of adapting an evidence-based parenting program for prevention of pediatric obesity and excess weight gain in primary care, submitted).

The FCU4Health is unique relative to other obesity interventions in that rather than solely targeting *knowledge* about national health guidelines (published by such organizations as the American Academy of Pediatrics and United States Department of Agriculture that require updating over time), it provides parents with the *skills and support* to implement the changes recommended by physicians, dieticians, and other members of the healthcare team. This aspect fills an important gap for families who earnestly agree to improve children’s diet, physical activity, sleep habits, or screen time while in the clinic but encounter difficulties in their regular involvement that lead to an abandonment of these efforts. It also increases the individualizability of the intervention to the needs of the family and reduces training time and the need for regular updates to program materials.

**Fig. 1:**
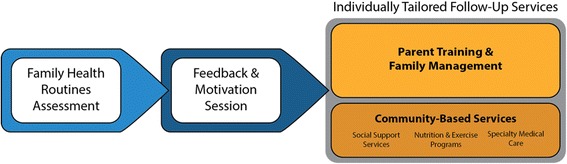
Steps and elements of the Family Check-Up for Health (FCU4Health) program in the Raising Healthy Children study

The core components of the FCU4Health mirror those of the original FCU: an ecological assessment of the family, a feedback and motivation session, and follow-up services that are individually tailored in content and dosage based on identified needs in the assessment (see Fig. 1). These follow-up services may include parenting modules for needs related to family management and/or care coordination with community-based services to address ecological needs [38]. Parenting modules follow *Everyday Parenting*, [45] a 12-module skills-based curriculum focusing on the three core areas of parenting and family management: relationship quality, positive behavior support, and monitoring and limit setting. The application of these steps in this trial is described in greater detail in the “Methods” section.

### Study design

#### Overview

We will test the FCU4Healtth with a type II hybrid effectiveness–implementation trial [46], which enables simultaneous evaluation of the clinical effectiveness and the delivery of the FCU4Health program using two different implementation strategies [47].

#### Implementation strategies and service delivery sites

The delivery model used by a given practice depends upon their programmatic, fiscal, and resource characteristics and needs [48]. Several approaches for the delivery of behavioral health services in primary care settings have been described [49]. In this trial, we partnered with three pediatric primary care agencies that serve high rates of low-income, ethnic minority families (about 60–65% Latino; 10% African American; 5–8% American Indian). Because pediatric primary care offices often do not have integrated behavioral health, arm 1 uses a strategy of coordinated care between primary care and an external behavioral health provider. We partnered with a large outpatient general pediatrics clinic associated with a children’s hospital. We also partnered with two agencies that are part of the nationwide network of 1250 Federally Qualified Health Centers (FQHCs). A feature of FQHCs is the integrated care model that typifies the medical home. In arm 2, we will test an integrated/co-located model of care. The FQHC network is crucial for embedding a family-centered approach to care because it serves youth and families at high sociodemographic risk and is intended to integrate services to reduce barriers and improve care. Partnering with these provider sites afforded the opportunity to compare FCU4Health implementation under two different real-life service delivery models. In both arms, behavioral health consultants are responsible for delivering the FCU4Health and coordinating care with other support services and resources.

#### Clinical effectiveness of the FCU4Health

Within the two implementation strategy arms, eligible families are randomized to receive either the FCU4Health or clinic services-as-usual plus community program information (see “Methods”). Primary clinical outcomes are child BMI and body composition. Secondary outcomes, hypothesized to mediate effects of the intervention on primary outcomes, are lifestyle behaviors (diet and nutrition, physical and sedentary activity, sleep), child self-regulation, and family management practices.

## Methods

### Study aims

#### Aim 1

Aim 1 was to finalize the adaptation of the FCU4Health, which was initially adapted and piloted in pediatric primary healthcare [50], based on input from a community advisory board (CAB) and partner clinics.

#### Aim 2

Aims 2 was to evaluate the effects of two delivery strategies: integrated/co-located care and coordinated care with referral. Using a mixed-methods approach applied to Proctor et al.’s [51, 52] taxonomy, we will evaluate the process of implementing the FCU4Health to inform, taking the program to scale.

- 2a. Evaluate fidelity over time to the FCU4Health using a validated observational rating system [53–55] and develop and test an automated fidelity coding system [56].
- 2b. Employ behavioral intervention costing methods [57] to evaluate the costs of installing and delivering the FCU4Health and conduct a cost–benefit analysis to evaluate the monetary benefits of program effects.
- 2c. Develop a plan to support scale-up and sustainment in collaboration with our CAB.

#### Aim 3

Aim 3 was to use pragmatic trial principles to test program effects on primary and secondary outcomes. Intervention effects on proximal outcomes will be tested as mediators on the distal outcomes of child BMI and body composition.

#### Aim 4

Aim 4 was to carefully track utilization in each aspect of the program, gather data concerning family’s service delivery preferences and satisfaction, and test a model of program implementation where family engagement mediates the relation between fidelity and clinical outcomes [58].

### Study participants, recruitment, and randomization

**Fig. 2:**
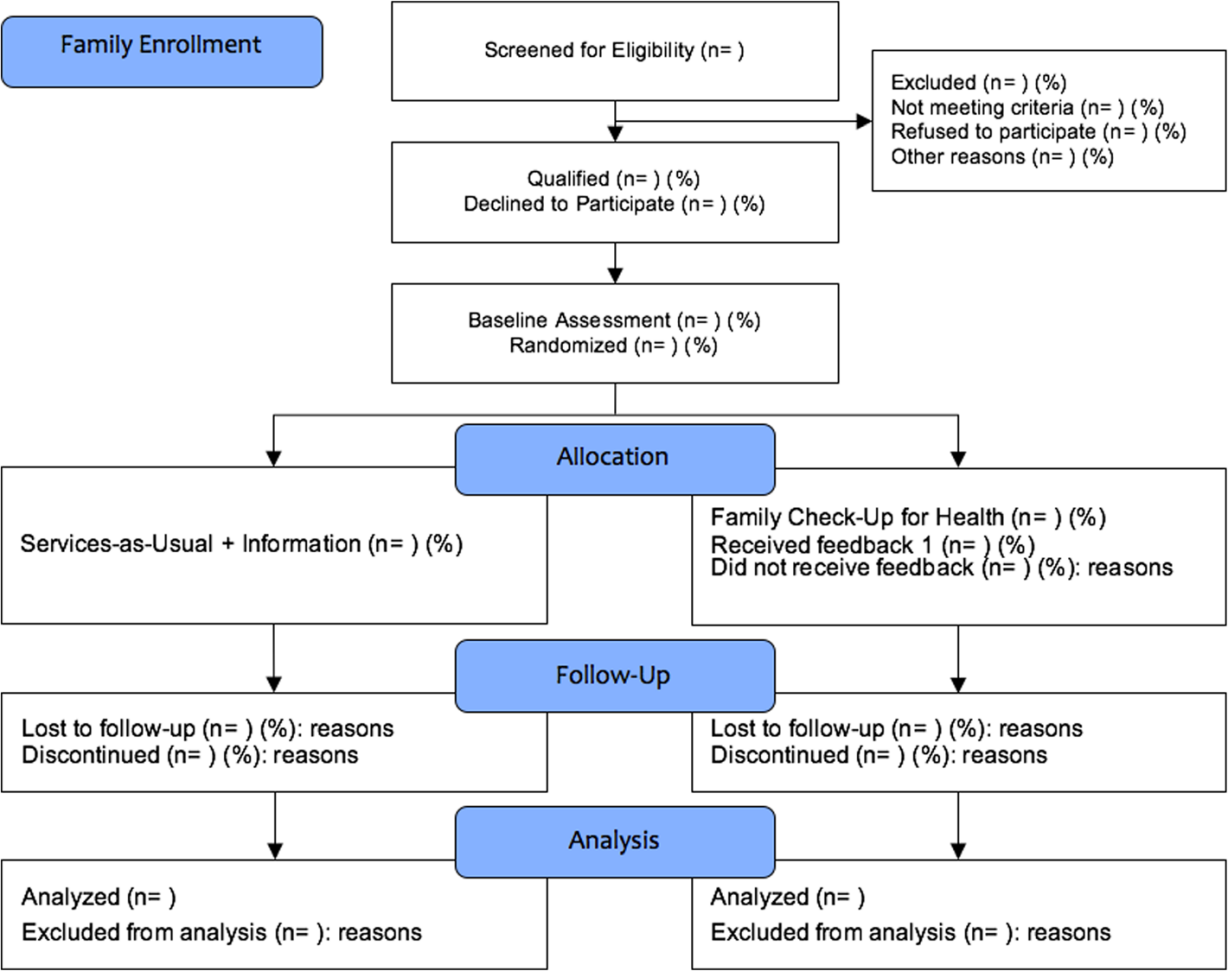
CONSORT flow diagram

Families with children 5.5 to 12 years of age with BMI ≥ 85th percentile for age and gender at study entry are eligible to participate. Participants will be identified during clinic well- and sick-child visits and through queries of the Electronic Health Record (EHR). Enrolled families will complete an ecological, family health routine assessment. After the first assessment, families will be randomly assigned to the FCU4Health (*n* = 200) or Services-as-Usual Plus Information condition (*n* = 150) using a stratified block randomized design by child gender, age, language (English, Spanish), and ethnicity. Assessing the family prior to randomization ensures double-blinding at baseline. Families in the intervention condition will participate in the FCU4Health program, as outlined in the next section, in addition to receiving usual care through their clinic. Families are compensated for completing assessments, but not for engaging in services. A CONSORT table is presented in Fig. 2.

### Family Check-Up 4 Health program

#### Delivery schedule

Previously, the FCU has used a health maintenance approach, in which parents receive periodic (annual) FCUs [38, 59]. Based on the US Preventive Services Task Force recommendations that overweight/obese youth should be provided with at least moderate treatment dosage to affect weight management [60], we designed a delivery schedule to achieve a minimum *targeted* dose of intervention between 25 and 50 h over 6 months (assessment and feedback at months 1, 3, and 6). Because of the tailored approach, families with greater need and those with greater motivation are expected to participate in more follow-up services [61].

#### FCU4Health family health routine assessment

The family health routine assessment is ecological and evaluates five broad domains—family health behaviors, child health behaviors, family well-being and support, child adjustment, and family management and relationships—using a multi-agent battery of caregiver- and child-reported questionnaires and a series of brief video-recorded family interaction tasks (FITs). Two versions of the assessment are used in this study: a full version at months 1, 6, and 12 (90 min to complete) and a brief version administered only at the 3-month assessment (30 min). The full version includes all questionnaires across the five broad domains and three FITs on the topics of health and diet goals, monitoring and limit setting of child health behaviors, and planning a fun physically active family activity (4 min each). The brief version includes only the questionnaires to caregiver and child pertaining to family health behaviors and child health behaviors and is intended as a low-burden opportunity to reinforce steps toward behavior change and address areas that have not yet improved or potentially shift focus to areas that have emerged during the first 3 months of intervention. Questionnaires are completed in English or in Spanish by caregivers and the child through a secure website accessed via a Wi-Fi enabled tablet. Pencil-and-paper versions are available if needed. FITs are recorded using the tablet and are uploaded to a cloud-based, HIPAA-compliant portal for secure storage, viewing, and scoring.

#### Feedback sessions

Feedback sessions follow the assessments in months 1, 3, and 6. The first feedback session begins with a discussion to understand (a) the caregivers’ perception of their needs; (b) their child’s health, adjustment, and obesogenic behaviors; and (c) the caregivers’ motivation to change parenting and family management practices in support of health behavior change. In the second and third feedbacks, the coordinator begins by checking in with the family about their progress, discussing barriers they are experiencing, and exploring the ways that the previous feedback and *Everyday Parenting* sessions were helpful for them in catalyzing and supporting healthy lifestyle behavior change. The coordinator presents the findings of the family health routine assessment using the Feedback Form, which summarizes where families are in the five areas of the assessment relative to age-based norms with a stoplight color scheme. Family strengths are represented in green, yellow indicates areas to monitor, and red accentuates areas in need of further support. The goals of this session are to (a) share assessment findings with family members regarding strengths and challenges, (b) engage in a motivation-enhancing discussion about promoting positive changes, and (c) provide a menu of resources and next steps for intervention. The first two feedback sessions lay out a plan for the follow-up services (i.e., *Everyday Parenting* sessions or referrals to community-based services) and use MI techniques to increase buy-in for the planned course of action. In the final feedback session (month 6), feedback and motivation focus on the progress the family has made and continuing engagement in community services to address areas in need of additional support.

#### *Everyday Parenting* sessions

In the FCU4Health, *Everyday Parenting* sessions are tailored to the family’s specific needs identified in the family health routine assessment and focus on a specific behavior change goal, such as setting limits on snacking between family meals or monitoring children’s sedentary and physical activity time. It is expected that families will engage in 8–16 sessions of *Everyday Parenting* over the 6-month intervention period. The focus is on supporting and maintaining children’s healthy behaviors. It is also expected that during these sessions, the FCU4Health coordinator will help the caregivers problem-solve challenges they have encountered in implementing recommended health behavior changes.

#### Community programs and support services

To address needs related to health behaviors (e.g., nutrition and physical activity programs), specialty medical needs (e.g., asthma, diabetes), and the social determinants of health that affect parents’ ability to support child health (e.g., employment, food and housing insecurity, insurance coverage), coordinators provide families with referrals to existing resources in the community. We will identify programs in collaboration with our partner agencies that are available at little or no cost to families. The intent is to leverage existing services to support families and to test whether the motivational aspects of the FCU4Health lead to increased engagement compared to families in usual care. A centralized repository of available programs and their requirements is stored in a Web-based hub, which can be updated by coordinators and other staff.

#### Phone-based coaching

In weeks where a face-to-face session is not scheduled or does not occur (e.g., cancelation, no show), the coordinator or a behavioral health support staff person (e.g., a community health worker) conducts a 15- to 30-min phone-based coaching session. The purpose is to maintain contact with the family and help problem-solve challenges, reinforce positive achievements, and continually address motivation to change and barriers to engagement. Additional information about the program is available in the FCU4Health protocol manual [62].

### Services-as-Usual Plus Information condition

Families randomized to the services-as-usual arm will receive care as usual from their primary care provider and any additional services offered by the agency, as well as a listing of the same community resources offered to families randomized to the FCU4Health arm.

### Measures

Additional information on measures of implementation and clinical effectiveness is presented in Tables 1 and 2, respectively.

**Table 1:** Implementation outcomes

Outcome variable(s)	Measure(s) and data collection procedures	Data source and reporter (when applicable)
Stakeholders: acceptability, feasibility, appropriateness, and sustainability	(1) Select scales of the Annual Survey of Evidence-Based Programs [113] (*α* > .75)	Survey (ST)
	(2) FCU4Health Stakeholder Survey: 11 open-ended questions, adapted from the Treatment Acceptability Rating Form, related to the relevance of the FCU4Health for obesity management, barriers and facilitators of the delivery of the program, and feasibility of this program from the perspective of stakeholders	
	(3) Program Sustainability Assessment Tool [114]: 8 domains (e.g., funding stability, organizational capacity) with 5 items each (e.g., “The program has sustained funding”) are rated on a 7-point Likert scale (1 = little or no extent, 7 = a very great extent) (*α* = .88)	
Caregivers: acceptability and appropriateness	(1) FCU4Health Caregiver Acceptability Interview, designed for and used in our pilot feasibility trial [50], consists of 11 open-ended questions pertaining to the relevance of the program components to the family’s efforts to manage weight, the acceptability of the program and its components, and the barriers and facilitators of participation	Interview (CG)
	(2) Treatment Acceptability Rating Form-Revised Short [115–117], adapted for FCU4Health: 10 items (e.g., “How likely is FCU4Health to make permanent improvements in your child’s health behaviors?”) rated on a 7-point Likert scale (1 = not at all, 7 = very) (*α* = .92) [115]	Survey (CG)
Reach	1- or 2-month sampling period to more closely approximate the number of families that require a service at any given point [63]	EHR data
Family service participation	(1) FCU4Health activities checklist (FACL) [118] (see description in the “Methods” section)	Administrative data and Survey (CO)
	(2) Community Resources: Engagement and Adequacy (CREA): adapted from an existing care coordination measure to assess the extent to which families engaged in community resources (e.g., emergency care, well-child visits, recreational and nutrition programs, mental healthcare, school services, financial services) and if help was needed to obtain the resource and whether those resources met their needs. Response options are as follows: “I didn’t need help”; “I needed help, but didn’t find it”; “I tried this, but it didn’t work”; “I’m still getting help”; and “I got help, and it worked”	Survey (CG)
	(3) Dosage of Engagement in Community Resources (DECR) [119]: created for this study to assess the amount of time spent in activities to support health behaviors assessed by asking the number of times among 7 response options (e.g., “once a month,” “2–3 times a week,” “2 times every day”) and then the duration of each instance of teach activity using a drop-down menu of min (e.g., 30) to h (e.g., 1, 3, and 8 h or more)	
Fidelity	(1) COACH observational rating system [54]: 5 dimensions of observable in-session coordinator skills: conceptual accuracy; observant and responsive to the families’ contexts and needs; actively structures session to optimize effectiveness; carefully teaches and provides corrective feedback; hope and motivation are generated. Each dimension contains exemplars (prescribed behaviors) and non-exemplars (proscribed behaviors) and is rated on a 9-point scale: 1–3 (needs work); 4–6 (competent work); 7–9 (excellent work) (ICC ≥ .73) [120]. Variability in fidelity ratings to feedback sessions have been associated with long-term changes in parenting skills and child behavioral outcomes [53–55]	Observational
	(2) Automated coding of fidelity is being developed within this study (aim 2b)	Automated coding
Costs and health economics	(1) Cost capture survey [57]	Survey (ST)
	(2) FCU4Health activities checklist (FACL) [118] (see description in the “Methods” section)	Survey (CO)
	(3) Electronic budgets	Administrative data
	(4) Health plan claims data	Administrative data

*CG* caregiver, *CO* FCU4Health coordinator, *ST* stakeholder, *EHR* electronic health record

**Table 2:** Clinical effectiveness and related child and family outcomes

Outcome variable(s)	Measure(s) and data collection procedures	Data source and reporter (when applicable)
Child weight and body composition	Portable electronic scale (Tanita SC-331SU) approved by the FDA for BMI and body composition in children (ages 5 to 18) and adults. Child BMI is standardized by sex and age according to the CDC growth reference data for children [121]	Anthropometric
Child dietary habits	NHANES Dietary Screener Questionnaire [122]: 9-point scale (0 = never, 8 = 6 or more times per day) to rate 3 items about fruit, vegetable, and fast-food choices (e.g., “In the past month, how often did you eat fruit?”) and 3 items regarding sugar-sweetened beverage choices (e.g., “In the past month, how often did you drink regular soda that contained sugar?”)	Survey (CG)
Family health routines and health behaviors	(1) Family Health Behaviors Scale [123]: 24 items rated on a 5-point scale (0 = almost never, 4 = nearly always; e.g., “I participate in physical activity with [child name]; [child name] sneaks food”). Caregiver ratings on this scale are sensitive to change and have been shown to predict child weight classification	Survey (CG, CH)
	(2) Sleep parenting routines: 6 items on a 5-point scale (0 = almost never, 4 = nearly always; e.g., [child name] goes to bed at about the same time each night). Items developed from existing measures, such as the Brief Infant Sleep Questionnaire [124]	
	(3) Media parenting routines: 6 items on a 5-point scale (0 = almost never, 4 = nearly always; e.g., “I keep track of [child name]’s screen-time”) and a single question on h per day of media use. Items were drawn from published studies and measures [125]	
Parenting and family management skills	Questionnaires encompass 3 domains of parenting and family management skills: (1) positive behavior support, (2) relationship quality, and (3) monitoring and limit setting. Each of these measures has been used in previous FCU trials and was found to have adequate reliability, internal consistency, and sensitivity to change	Survey (CG, CH)
	Caregiver: 5-point scale (0 = never, 4 = very often) on the domains of incentives and encouragement (4 items; e.g., “Gave [child name] a hug, kiss, or kind word”) [126]; proactive parenting (7 items; e.g., “Plan for ways to prevent problem behavior”) [127], parent–child conflict (10 items; e.g., “[child name] gets angry at me easily”); family conflict (5 items; e.g., “We got angry at each other”) [126]; quality time (6 items; e.g., “Involve [child name] in household chores”) [127], parent warmth (5 items; e.g., “If upset, [child name] seeks comfort from me”) [128]; family routines (7 items; e.g., “Check to see if [child name] has homework”); limit setting (7 items, e.g., “Speak calmly with [child name] when you were upset with him/her”), negative parent behavior (5 items; e.g., “Criticize [child name]”) [127]; and a single question on h per day of unsupervised time	
	Child: 4 items on incentives and encouragement (e.g., “Praised you or complimented you for something you did well”), using a 5-point scale (0 = never, 4 = very often); 4-item questionnaire on family conflict (e.g., “I got my way by getting angry”), using a 7-point scale (0 = never, 6 = 8+ times) [126]	
	Family interaction task (FIT) observational coding system [129]: the recorded family interactions are scored for caregiver(s) behaviors in the domains of relationship quality, positive behavior support, and monitoring and limit setting, as well as demonstrated knowledge of children’s health behaviors (e.g., age-appropriate physical activity duration and dietary guidelines). Child behaviors and emotional adaptation are rated. Each domain is rated for parent’s skill/knowledge on a 5-point scale (1 = low, 5 = high) for each interaction task independently	Observational
Child self-regulation	Caregiver: 13-item survey (e.g., “[child name] is able to resist laughing or smiling when it isn’t appropriate”) adapted from the Children’s Behavior Questionnaire [130], using a 5-point scale (1 = almost always untrue, 5 = almost always true)	Survey (CG, CH)
	Child: 16-item questionnaire (e.g., “I pay close attention when someone tells me how to do something”) adapted from the Early Adolescent Temperament Questionnaire [131], using a 6-point scale (1 = always untrue, 5 = always true)	
Weight-related stigma	Perception of Teasing Scale [132]: children will use a 5-point scale (1 = never/not upset, 4 = very often/extremely upset) to rate the frequency of 3 events pertaining to weight-related stigma and describe their level of distress associated with these items (e.g., “People made fun of you because you were heavy”; “How upset does this make you?”)	Survey (CH)
Body image	Body Image Scale for Children: a pictorial scale using body pictures representing standardized percentile curves of BMI for boys and girls, separately; good reliability and evidence of validity with children aged 7 to 12 [133]; proxy for satisfaction and a measure of potential adverse effects of participation in the program/study	Survey (CH)
Quality of life	Pediatric Quality of Life Inventory [134]: 23 items in four categories: physical functioning, emotional functioning, social functioning, and school functioning	Survey (CG, CH)
	Caregivers: 5-point scale (0 = never, 4 = almost always) to rate items (e.g., “[child name] feels afraid or scared”; “[child name] gets along with other children”)	
	Children: 3-point scale (0 = not at all, 2 = somewhat, 4 = a lot) to rate items (e.g., “It is hard for me to run”; “It is hard for me to pay attention in school”) adjusted for 2 age groups: 5–7 years old and 8–12 years old	
Satisfaction with care	(1) Family Check-Up Caregiver Service Satisfaction Survey: 9 items rated on a 4-point scale (0 = strongly disagree, 4 = strongly agree) adapted from the Client Satisfaction Questionnaire [135] to be specific to parent training programs. This was developed for use with the original FCU conducted in community mental health clinics [89] (*α* = .95) in that trial	Survey (CG)
	(2) Parent Experience of Assessment Survey (PEAS) [136]: 3 of the 5 subscales (parent–coordinator collaboration, systemic awareness, and negative feelings) with 15 total items rated on a 5-point scale (1 = strongly disagree, 5 = strongly agree) (*α* > .75)	
Child adjustment	Strengths and Difficulties Questionnaire [137]: caregivers and children rate 5 items each on a 3-point scale (0 = not true, 1 = somewhat true, 4 = very true) on the conduct problems (e.g., “I/[child name] often lose(s) temper”), hyperactivity (e.g., “I am/[child name] is constantly fidgeting or squirming”), pro-social behavior (e.g., “I am/[child name] is considerate of other people’s feelings”), and emotional problems (e.g., “I am/[child name] is often unhappy, depressed, or sad”) subscales	Survey (CG, CH)

*CH* child, *CG* caregiver, *CO* FCU4Health coordinator, *ST* stakeholder, *EHR* electronic health record

#### Implementation

To assess the *acceptability*, *feasibility*, *appropriateness*, and *sustainability* of each component of the FCU4Health (e.g., training, program costs, content, delivery), an agency stakeholder battery will be electronically administered to at least 30 agency stakeholders (approximately ten from each agency) at two times during the study: 4 months into the FCU4Health delivery and when all families enrolled in the study complete the entire 6-month FCU4Health protocol. Families in the FCU4Health arm will rate the acceptability and appropriateness of the program via an electronically administered survey at months 6 and 12. Additionally, 20% of families in the FCU4Health arm will be randomly selected and interviewed by study staff via phone.

Study enrollment and participation data will be used to evaluate the *reach* of the program in each agency by tracking both the proportion of eligible families referred to the study and the number randomized to the FCU4Health condition that engage in the program compared with the total number of eligible families [63].

We will assess the *fidelity* of coordinators to the FCU4Health using an established observation rating system that defines high-quality care and was associated with clinical improvement in studies of the original FCU [53, 54]. A trained coding team will code video or audio recordings of the first FCU4Health feedback session from every family (*N* = 200), and a subset of 80 families will be randomly selected at study entry, balanced by coordinator, for longitudinal coding of all three FCU4Health feedback sessions to assess drift (*N* = 160 additional sessions). In addition, we will develop an automated coding system based on an existing, validated automated coding system for motivational interviewing. Automated fidelity coding will be built from previous methodologies developed for coding fidelity to MI (presence of complex reflections, open-ended questions) [56, 64, 65] and family interventions [66, 67]. Machine-generated fidelity codes have been found to be reliable with human coding across multiple studies [56, 64–66, 68, 69]. Initial algorithm training will use existing FCU feedback session recordings and the FCU fidelity codebook. Automated coding involves a computational “pipeline” of both speech signal processing and machine learning of the audio recordings of the conversations during the sessions, including (1) voice activity detection, (2) diarization (i.e., how many speakers are in session and when is each talking?), (3) role identification (i.e., who is the provider?), (4) automatic speech-to-text transcription, and (5) machine learning models to predict fidelity codes, using lexical (i.e., words) and paralinguistic features as inputs.

*Cost* data collection is multi-method in order to perform analyses from multiple perspectives (e.g., agency, payor, family). Study staff report activities weekly on an electronically administered survey. FCU4Health coordinators complete a brief survey after each contact with study families (in the intervention arm) to capture the staff involved and the number of hours spent. Electronic budgets track program spending and will be used to prospectively separate costs associated with implementation from those specifically related to research (e.g., participant reimbursement, data analysis software), start-up (e.g., training), and ongoing costs (e.g., consultation with FCU4Health developers, technical assistance, travel for home visitation). At each assessment, families will be asked to provide a release for the study team to obtain health plan claims data, which will be used to identify relevant health services utilization and associated costs.

Critical to the study’s implementation aims, such as the calculation of reach and cost, is the careful tracking of participation in the various components of the program. Using methods developed and tested in previous trials, the FCU4Health coordinators and other agency staff will track all family contacts and record the delivery location, travel time, details of participating family members, the type of the FCU4Health session, content areas covered, and referrals. At each assessment, caregivers will report on their engagement in community resources, how well these resources met their needs, and the amount of time spent in activities to support health child and family behaviors.

#### Effectiveness

At each assessment, the child and caregiver(s) will be weighed using a portable electronic scale. *BMI* is the primary study outcome because it indicates cardiometabolic disease progression [70–72]. However, improvement in *body composition* in the absence of weight loss can indicate a healthy change in the ratio of fat to lean muscle, which often accompanies significant changes in diet and physical activity and results in metabolic improvement with enduring benefits [73, 74]. At each assessment point, caregivers and children report on *health-promoting family behaviors*; *mealtime, sleep, and media routines*; *child and family physical activity habits*; *child food and beverage choices*; and *use of community support services*. The FITs are used for observational assessment of parenting and family management skills using the validated Coder Impressions Inventory [75] (Dishion T, Hogansen J, Winter C, Jabson J. The coder impressions inventory, unpublished manual). Although not central to our aims, we will also assess several other constructs related to pediatric obesity: *weight-related stigma*, *body image*, *quality of life*, *child behavioral and emotional adjustment*, and *caregiver satisfaction with services*.

### Data analysis

#### Overview

Analyses focus on implementation data from the families randomized to the FCU4Health. Data from families receiving services as usual will be used in aims 2b and 3. Before data analysis, we will test the psychometric properties (i.e., distribution, reliability) of the measures, conduct a sequence of nested multiple-group confirmatory factor analyses [76] to ensure measurement invariance across language and ethnicity, and apply state-of-the-science data reduction techniques (confirmatory factor analysis, weighted regression scores) by creating multi-indicator, multi-informant construct variables when applicable. These techniques improve measurement properties and result in fewer contrasts, reducing type I error [77]. We expect the attrition rate across waves to be 10–15% based on retention rates for local studies with highly mobile, hard-to-track samples [78, 79]. We will adjust for missing data due to attrition using multiple imputation [80] and full information maximum likelihood [81]. We will perform sensitivity analyses to check the non-response mechanism [82] and attrition analyses to detect differential attrition rates and attrition by group interaction on sociodemographic and pretest variables that may pose a threat to the validity or the equity of the findings [83]. When missingness is not ignorable, we will use complex models to obtain parameter estimates [84]. To estimate power, we used Optimal Design [85] and Monte Carlo simulations in Mplus [84] and referred to relevant publications. All power analyses used 1 − *β* = .80 and *α* = .05 using two-tailed tests. For mediation models, we applied simulation in Mplus for estimating the power of path coefficients and referred to a work by Fritz and MacKinnon [86] for examining significance of mediation effects.

For qualitative data analysis, stakeholder and caregiver interviews and CAB discussions will be audio recorded, transcribed, and then coded using a directed content analysis approach [87] by using existing theory, prior results, and empirical frameworks to develop a coding scheme. Codes will be refined using a subsample of interviews, and then a team of trained coders will code full transcripts. Twenty percent of interviews will be double coded in order to calculate reliability [88]. Disagreements in coding will be resolved via consensus.

#### Aim 1

Results of the adaptation activities are largely descriptive and involve the use of qualitative data from the CAB small group discussions to describe the process and content of adaptations to the program during the course of the trial.

#### Aim 2

In addition to the specific outcomes in aims 2a–2c, we will compare the two arms on stakeholder and caregiver acceptability using repeated-measures analysis of variance (ANOVA) to analyze scores of approximately 30 stakeholders across two time points. We have sufficient power to detect a medium effect for this analysis (*f* ≥ .32). Concerning the variance of caregiver acceptability ratings between groups or over time, we have sufficient power to detect a medium effect (*f* ≥ .28). Latent growth curve (LGC) analyses will be used to determine group differences in the trajectory over time. The estimated power for this analysis is *β* = 0.12.

#### Aim 2a: fidelity

We will calculate the mean score, interrater correlation coefficient, and internal consistency of the observational ratings. Using a mixed-effects model with random effects for families and a fixed-effects model, we will compare scores from prior FCU trials [53–55, 89] (Smith JD, Dishion TJ, Rudo-Stern J, Stormshak EA, Brown K, Ramos K, Thornton N, Shaw DS, Wilson MN, , A quasi-experimental study of the sensitivity and efficiency of observationally assessing fidelity, submitted) to those from the current trial. We have power to detect a small effect of *d* ≥ .18. Second, we will use the LGC analysis to (1) evaluate drift in fidelity [53] (power to detect a small effect of *d* ≥ .16 for *μ*_slope_) with the sample size of 80 families and 240 feedback sessions and (2) assess the relation between variability in fidelity on program effects (power to detect a small effect of *β* ≥ .10 for *μ*_slope_ on outcomes). To evaluate fidelity using automated coding, we will conduct the same analyses as above but with all 600 feedback sessions. Even when controlling for imperfect reliability, we have power to detect a small effect of *d* ≥ .10 for comparison to previous trials, a small effect of *d* ≥ .11 for *μ*_slope_, and a small effect of *β* ≥ .08 for *μ*_slope_ on outcomes.

#### Aim 2b: costs

An ingredient-based cost analysis procedure will be employed to estimate FCU4Health installation and continued implementation costs. Using an activity-based costing approach will allow us to value activities both locally and from national data sources (e.g., US Bureau of Labor Statistics), providing estimates relevant for scale-up in new pediatric care settings nationwide. First, we will conduct a budget impact analysis (BIA) of the cost to install the program (i.e., prior to enrollment and provision of care). BIA provides an estimate of the financial consequences of adopting a new intervention from the perspective of the entity financing the intervention [90]. Second, a multi-perspective cost–benefit analysis will be undertaken to capture the potential impact of the FCU4Health on different key stakeholders (i.e., payor, hospital, family). The fiscal models will be structured to estimate monetary benefits associated with program effects. For significant program effects on the outcome measures in aims 1 and 2, we will calculate the total benefit as a function of the size of the effect (*Q*) and the price per unit (*P*) for each year (*y*). This value can be simply represented as *B*_*y*_ = *Q*_*y*_ × *P*_*y*_. Effects at 6 and 12 months will be adjusted using a discount rate to accommodate the impact of time on our estimates. To avoid overlap or *double-counting* benefits, we will employ a weighted average approach of outcomes [91]. We will calculate the average based on the total effect sizes for each outcome in order to estimate the benefits from a single monetary source. While point estimates often represent the bottom line for such an evaluation (e.g., a return-on-investment amount), a confidence interval that incorporates different values of model input that might feasibly occur is more appropriate given the various model assumptions used [92]. A common model input that could take a range of values represents economic uncertainty (i.e., the discounting rate) [93]. In addition to representing the variation that might be expected due to sampling, we will also consider a range of estimates due to anticipated variation in program characteristics. We will consider how key aspects of the delivery system, such as facility costs or provider salary, might vary with new installations.

#### Aim 2c: sustainability

The sustainability plan will be largely descriptive and involve the synthesis of quantitative and qualitative data across implementation outcomes, caregiver satisfaction survey results, and CAB guidance. We will use a mixed-methods data analytic approach (QUANT = QUAL) [94] to develop the sustainability plan.

#### Aim 3

We will apply intent-to-treat analyses, using data from all participants who are randomly assigned to the two arms. Our unbalanced design (200 FCU4Health, 150 services-as-usual) only slightly affects statistical power [95]. The primary outcomes are the distal effects on child weight status and body composition and effects on more proximal mediators, including healthy lifestyle (dietary and physical activity habits), child self-regulation, parenting variables, and use of community support services. The LGC analysis for the continuous BMI and body composition measures will be used to compare the trajectories of the two conditions over time analyzed with random-effects models [96]. Specifically, we will estimate whether individual changes (i.e., growth trajectories) over time vary from person to person and whether inter-individual variation is systematically related to intervention assignment. For comparing FCU4Health and control arms using analysis of covariance (ANCOVA), we have power to detect a mean difference with a small-to-medium effect size (*d* ≥ .25; odds ratio ≥ 1.5, assuming the base rate of the control group is 30%). This means we have power to detect the size of effect on BMI found in previous trials with children and early adolescents, which varies significantly from small to medium (range: *d* = .20–.40) across intervention types [97–100] but is large (*d* ≥ .50) for family-centered behavioral programs [101, 102]. For the growth models (repeated-measures analysis using three or four data points), we have power to detect a small effect on the trajectory (*β* = .12 or *η*^2^ ≤ 2%). LGC modeling has better power than the two-time point ANCOVA for detecting treatment effects in a randomized design [103]. Second, we will examine whether program effects on the distal outcomes are transmitted through the program effects on the proximal variables using mediation analyses, controlling for baseline measures of the mediator and outcome. Bootstrapping will be used to form confidence intervals to test for mediation effects, which has been shown to have better power [86, 104]. We have power to detect a mediated effect if the two paths each have a small-to-medium effect (*β* values ≥ .14).

Baseline weight, ecological risk, minority status, poverty status, and child gender will be included as covariates and tested as potential moderators. Moderation of program effects will be examined by adding a program by moderator interaction in the models of primary outcomes and mediators one at a time. For significant interactions, post hoc probing of simple effects (i.e., effects at different values of the moderator) will be conducted, following Aiken and West [105]. Accounting for the imperfect reliability, we have power to detect moderation effects that account for *R*^2^ ≤ 3% of the outcome [106].

We will examine whether it is necessary to account for site and coordinator effects by calculating the design effect metric [107]. If significant, we will modify our statistical plans and utilize multi-level models with site or coordinator as the cluster variable.

#### Aim 4

To determine differences in family participation between conditions, we will use the Wilcoxon rank-sum tests. We have sufficient power to detect a medium effect for this analysis (*f* ≥ .30). Next, we will use path analysis in structural equation modeling to test a model of program implementation where family engagement mediates the relation between fidelity and clinical outcomes [58]. Using automated fidelity data on the FCU4Health condition sample (*N* = 200), we have power to detect a mediated effect if the two paths each has a small-to-medium effect (*β* values ≥ .21).

### Study status

At the time this manuscript was submitted for publication, the study was underway. We are planning for our fourth CAB meeting; initial adaptation of the program and assessment is complete; interviewers, coordinators, and other program staff have been trained; and recruitment is in progress at all three agencies. We have also begun formative work on the development of the automated fidelity coding system using data from previous FCU trials.

## Discussion

Similar to some previously published study protocols in this journal, our type II hybrid effectiveness–implementation trial blends pragmatic trial and implementation science frameworks. However, our protocol is somewhat unique to the hybrid trial design described by Curran et al. [46] because the two implementation strategies differ on the pragmatic–explanatory trial continuum [108] in which the integrated/co-located care arm of the trial is more pragmatic compared to the coordinated care with referral condition, which is more explanatory. However, both conditions are far more pragmatic than they are explanatory—as would be expected for a hybrid trial. This was done to speed translation given that the effectiveness of the adapted FCU4Health program was partially supported by the large existing corpus of evidence supporting the original FCU to both change the purported mediators as well as the primary child outcomes [44]. Additionally, the request for applications for the Childhood Obesity Research Demonstration Projects 2.0 indicated preference for such a design [109].

One of the primary challenges we have encountered since beginning this study relates to working with healthcare organizations. Context is always critical to implementation research, and differences are magnified when attempting to implement a complex behavioral intervention where, previously, a similar program did not exist. The partnerships between the research team and the community sites are critical for success [110], and we have been able to remain successful due to the strength of our partnership, but administrative, personnel, and other issues required extensive meetings, which protracted our planned preparation time from 6 months to nearly 12 months. Many of the issues that were raised could not have been avoided. In part, the extended time was due to variation within our partner clinics that necessitated meetings as well as continually garnering buy-in from leadership and staff that would deliver the FCU4Health.

In conclusion, this study was designed to address a larger issue in the field of pediatric obesity management—the lack of penetration of evidence-based programs into the primary healthcare system. The B rating for family-centered weight management interventions for children aged 6 to 12 years with overweight and obesity by the US Preventive Services Task Force [111] provided a financial avenue for such interventions in primary care. This study was aimed at testing and evaluating the many factors that comprise the struggle of making a complex, evidence-based program work in the real world [112]. Achieving wide-scale adoption of the FCU4Health, if found to be effective, would impact the pediatric obesity epidemic in a way that could simultaneously address a number of the factors that result in healthcare disparities for chronic conditions.

## Additional file

Additional file 1: Notice of award. Notice of award from CDC for year 1. (PDF 245 kb)

## Abbreviations

ANCOVA: Analysis of covariance
ANOVA: Analysis of variance
BMI: Body mass index
CAB: Community advisory board
FCU: Family Check-Up
FCU4Health: Family Check-Up 4 Health
FQHC: Federally Qualified Health Center
LGC: Latent growth curve
MI: Motivational interviewing
