# Supplementary material for: An individually tailored family-centered intervention for pediatric obesity in primary care: study protocol of a randomized type II hybrid effectiveness–implementation trial (Raising Healthy Children study)
Source: Implement Sci. 2018 Jan 15;13:11. doi: 10.1186/s13012-017-0697-2 (PMC5769381; doi:10.1186/s13012-017-0697-2)
Supplement: Additional file 1: — Notice of award. Notice of award from CDC for year 1. (PDF 245 kb) [file 13012_2017_697_MOESM1_ESM.pdf]

*RESEARCH DEMONSTRATION COOPERATIVE AGREEMENTS* **Issue Date:** 05/26/2016

Department of Health and Human Services
Centers for Disease Control and Prevention
NATIONAL CENTER FOR CHRONIC DISEASE PREV AND HEALTH PROMO

**Grant Number:** 1U18DP006255-01
**FAIN:** U18DP006255

**Principal Investigator(s):**
Cady Berkel (contact), PHD
JUSTIN D SMITH, PHD

**Project Title:** An Individually Tailored, Family-Centered Intervention for Childhood Obesity: Connecting Services in Pediatric Primary Healthcare, the Home, and the Community

Ms. Mosley, Lisa
Executive Director for Research Operations
PO Box 876011
Tempe, AZ 852876011

**Award e-mailed to:** ASU.awards@asu.edu

**Budget Period:** 06/01/2016 – 05/31/2017
**Project Period:** 06/01/2016 – 09/29/2018

Dear Business Official:

The Centers for Disease Control and Prevention hereby awards a grant in the amount of $1,714,950 (see “Award Calculation” in Section I and “Terms and Conditions” in Section III) to ARIZONA STATE UNIVERSITY in support of the above referenced project. This award is pursuant to the authority of PHS Act,Sec 1706,42 USC 300u-5,as amended;Sec2(d),PL 98-551 and is subject to the requirements of this statute and regulation and of other referenced, incorporated or attached terms and conditions.

Acceptance of this award including the “Terms and Conditions” is acknowledged by the grantee when funds are drawn down or otherwise obtained from the grant payment system.

If you have any questions about this award, please contact the individual(s) referenced in Section IV.

Sincerely yours,

Roslyn Curington
Grants Management Officer
Centers for Disease Control and Prevention

Additional information follows

## SECTION I – AWARD DATA – 1U18DP006255-01

**Award Calculation (U.S. Dollars)**

| | |
|---|---|
| **Salaries and Wages** | $458,302 |
| **Fringe Benefits** | $119,124 |
| **Personnel Costs (Subtotal)** | $577,426 |
| **Supplies** | $57,558 |
| **Travel Costs** | $27,332 |
| **Other Costs** | $211,636 |
| **Consortium/Contractual Cost** | $342,164 |

| | |
|---|---|
| **Federal Direct Costs** | $1,216,116 |
| **Federal F&A Costs** | $498,834 |
| **Approved Budget** | $1,714,950 |
| **Federal Share** | $1,714,950 |
| **TOTAL FEDERAL AWARD AMOUNT** | $1,714,950 |

| | |
|---|---|
| **AMOUNT OF THIS ACTION (FEDERAL SHARE)** | $1,714,950 |

Recommended future year total cost support, subject to the availability of funds and satisfactory progress of the project.

02 $498,834

**Fiscal Information:**

| | |
|---|---|
| **CFDA Number:** | 93.535 |
| **EIN:** | 1860196696A1 |
| **Document Number:** | 16DP006255 |

| IC | CAN | 2016 | 2017 |
|---|---|---|---|
| DP | 93905BM | $1,714,950 | $498,834 |

| SUMMARY TOTALS FOR ALL YEARS | | |
|---|---|---|
| **YR** | **THIS AWARD** | **CUMULATIVE TOTALS** |
| 1 | $1,714,950 | $1,714,950 |
| 2 | $498,834 | $498,834 |

Recommended future year total cost support, subject to the availability of funds and satisfactory progress of the project

**CDC Administrative Data:**
**PCC**: / **OC**: 4141 / **Processed**: CURINGTONR 05/25/2016

## SECTION II – PAYMENT/HOTLINE INFORMATION – 1U18DP006255-01

For payment information see Payment Information section in Additional Terms and Conditions.

INSPECTOR GENERAL: The HHS Office Inspector General (OIG) maintains a toll-free number (1-800-HHS-TIPS [1-800-447-8477]) for receiving information concerning fraud, waste or abuse under grants and cooperative agreements. Information also may be submitted by e-mail to hhstips@oig.hhs.gov or by mail to Office of the Inspector General, Department of Health and Human Services, Attn: HOTLINE, 330 Independence Ave., SW, Washington DC 20201. Such reports are treated as sensitive material and submitters may decline to give their names if they choose to remain anonymous. This note replaces the Inspector General contact information cited in previous notice of award.

## SECTION III – TERMS AND CONDITIONS – 1U18DP006255-01

This award is based on the application submitted to, and as approved by, CDC on the above-titled project and is subject to the terms and conditions incorporated either directly or by reference in the following:

a. The grant program legislation and program regulation cited in this Notice of Award.
b. The restrictions on the expenditure of federal funds in appropriations acts to the extent those restrictions are pertinent to the award.
c. 45 CFR Part 74 or 45 CFR Part 92 as applicable.
d. The HS Grants Policy Statement, including addenda in effect as of the beginning date of the budget period.
e. This award notice, INCLUDING THE TERMS AND CONDITIONS CITED BELOW.

This award has been assigned the Federal Award Identification Number (FAIN) U18DP006255. Recipients must document the assigned FAIN on each consortium/subaward issued under this award.

**Treatment of Program Income:**
Additional Costs

---

**SECTION IV – DP Special Terms and Conditions – 1U18DP006255-01**

Funding Opportunity Announcement (FOA) Number: **DP16-004**
Award Number: **U18DP006255-01**
Award Type: **Cooperative Agreement**
Applicable Regulations: 45 Code of Federal Regulations (CFR) Part 75, Uniform Administrative Requirements, Cost Principles, and Audit Requirements for HHS Awards

## 45 CFR Part 75 supersedes regulations at 45 CFR Part 74 and Part 92

**AWARD INFORMATION**

**Incorporation**: The Centers for Disease Control and Prevention (CDC) hereby incorporates Funding Opportunity Announcement number DP16-004, entitled Childhood Obesity Research Demonstration 2.0, and application dated January 27, 2016, as may be amended, which are hereby made a part of this Research award hereinafter referred to as the Notice of Award (NoA). The Department of Health and Human Services (HHS) grant recipients must comply with all terms and conditions outlined in their NoA, including grants policy terms and conditions contained in applicable HHS Grants Policy Statements, and requirements imposed by program statutes and regulations, Executive Orders, and HHS grant administration regulations, as applicable; as well as any requirements or limitations in any applicable appropriations acts. The term grant is used throughout this notice and includes cooperative agreements.

Note: In the event that any requirement in this Notice of Award, the Funding Opportunity Announcement, the HHS GPS, 45 CFR Part 75, or applicable statutes/appropriations acts conflict, then statutes and regulations take precedence.

**Approved Funding:** Funding in the amount of $**1,714,950** is approved for the Year 01, budget period, which is **June 1, 2016** through **May 31, 2017**. All future year funding will be based on satisfactory programmatic progress and the availability of funds.

Note: Refer to the Payment Information section for draw down and Payment Management System (PMS) subaccount information.

**Award Funding:** Not funded by the Prevention and Public Health Fund

**Summary Statement Response Requirement**: The review comments on the strengths and weaknesses of the proposal are provided as part of this award. A response to the weaknesses in these statements must be submitted to and approved, in writing, by the Grants Management Specialist/Grants Management Officer (GMS/GMO) noted in the Staff Contacts section of this

NoA, no later than 30 days from the budget period start date. Failure to submit the required information by the due date, **July 1, 2016**, will cause delay in programmatic progress and will adversely affect the future funding of this project.

**Research Plan**: A revised Research Plan should be submitted the Grants Management Specialist within 30 days of the award, due date **July 1, 2016**

**Expanded Authorities:** The grantee is not permitted expanded authorities in the administration of the award.

**Program Income**: Any program income generated under this grant or cooperative agreement will be used in accordance with the Addition alternative

Addition alternative**:** Under this alternative, program income is added to the funds committed to the project/program and is used to further eligible project/program objectives.

Note: The disposition of program income must have written prior approval from the GMO

## FUNDING RESTRICTIONS AND LIMITATIONS

**Program Restrictions**: Applicants are advised that any activities involving standard information collection (i.e., surveys, questionnaires, data requests, etc.) from 10 or more non-federal individual/entities are subject to Paperwork Reduction Act (PRA) requirements and may require the CDC to coordinate an OMB/PRA approval request.

Funds will be restricted until:

IRB is obtained an 10% funding restriction in the amount of **$171,495** is placed on this award pending receipt of Certification of IRB approval of the projects(s)/protocol(s) for research under FOA #DP16-004. Human subject's research is prohibited until this Certification documentation has been obtained, submitted and approved by the Grants Management Specialist .
Human Subjects Education Requirement documentation is provided. Documentation of key personnel and other significant contributors involved in the design or conduct of research should be provided within 30 days of award by **July 1, 2016**, demonstrating completion of an education program in the protection of human subjects.

**Indirect Cost:** Indirect Cost Rates are based on the rate agreement dated **June 15, 2015**. The rates in this agreement are to be used for remainder of the competitive segment in accordance with 45 CFR Part 75. An indirect cost/facilities and administration rate is awarded based on the latest rate agreement dated **June 15, 2015**. Indirect cost/facilities and administration rates for subcontracts will be treated in the same manner as those for the awardee, if the subcontractor is covered by 45 CFR Part 75.

| Type | Effective Date From | Effective Date To | Rate (%) | Location(s) Applicable To |
|---|---|---|---|---|
| Predetermined | July 1, 2014 | June 30, 2016 | 54.5 | N/A |

**Cost Limitations:**

A. Cap on Salaries (Sec. 203): None of the funds appropriated in this title shall be used to pay the salary of an individual, through a grant or other extramural mechanism, at a rate in excess of Executive Level II.

Note: The salary rate limitation does not restrict the salary that an organization may pay an individual working under an HHS contract or order; it merely limits the portion of that salary that may be paid with Federal funds.

B. Gun Control Prohibition (Div. H, Title II, Sec. 217): None of the funds made available in this title may be used, in whole or in part, to advocate or promote gun control.

C. Lobbying Restrictions (Sec. 503):

- 503(a): No part of any appropriation contained in this Act or transferred pursuant to section

4002 of Public Law 111-148 shall be used, other than for normal and recognized executive-legislative relationships, for publicity or propaganda purposes, for the preparation, distribution, or use of any kit, pamphlet, booklet, publication, electronic communication, radio, television, or video presentation designed to support or defeat the enactment of legislation before the Congress or any State or local legislature or legislative body, except in presentation of the Congress or any State or local legislature itself, or designed to support or defeat any proposed or pending regulation, administrative action, or order issued by the executive branch of any State or local government itself.

- 503 (b): No part of any appropriation contained in this Act or transferred pursuant to section 4002 of Public Law 111-148 shall be used to pay the salary or expenses of any grant or contract recipient, or agent acting for such recipient, related to any activity designed to influence the enactment of legislation, appropriations, regulation, administrative action, or Executive order proposed or pending before the Congress or any State government, State legislature or local legislature or legislative body, other than normal and recognized executive legislative relationships or participation by an agency or officer of an State, local or tribal government in policymaking and administrative processes within the executive branch of that government.

- 503(c): The prohibitions in subsections (a) and (b) shall include any activity to advocate or promote any proposed, pending or future Federal, State or local tax increase, or any proposed, pending, or future requirement or restriction on any legal consumer product, including its sale of marketing, including but not limited to the advocacy or promotion of gun control.

For additional information, see Additional Requirement 12 at http://www.cdc.gov/grants/additionalrequirements/index.html and Anti Lobbying Restrictions for CDC Grantees at http://www.cdc.gov/grants/documents/Anti-Lobbying_Restrictions_for_CDC_Grantees_July_2012.pdf

D. Needle Exchange (Sec. 521): Notwithstanding any other provision of this Act, no funds appropriated in this Act shall be used to carry out any program of distributing sterile needles or syringes for the hypodermic injection of any illegal drug.
E. Blocking access to pornography (Sec. 526): (a) None of the funds made available in this Act may be used to maintain or establish a computer network unless such network blocks the viewing, downloading, and exchanging of pornography; (b) Nothing in subsection (a) shall limit the use of funds necessary for any Federal, State, tribal, or local law enforcement agency or any other entity carrying out criminal investigations, prosecution, or adjudication activities.

**Rent or Space Costs**: Grantees are responsible for ensuring that all costs included in this proposal to establish billing or final indirect cost rates are allowable in accordance with the requirements of the Federal award(s) to which they apply, including 45 CFR Part 75, Uniform Administrative Requirements, Cost Principles, and Audit Requirements for HHS Awards. The grantee also has a responsibility to ensure sub-recipients expend funds in compliance with applicable federal laws and regulations. Furthermore, it is the responsibility of the grantee to ensure rent is a legitimate direct cost line item, which the grantee has supported in current and/or prior projects and these same costs have been treated as indirect costs that have not been claimed as direct costs. If rent is claimed as direct cost, the grantee must provide a narrative justification, which describes their prescribed policy to include the effective date to the assigned Grants Management Specialist (GMS) identified in the CDC Contacts for this award.

**Trafficking In Persons**: This award is subject to the requirements of the Trafficking Victims Protection Act of 2000, as amended (22 U.S.C. Part 7104(g)).

**Cancel Year**: 31 U.S.C. Part 1552(a) Procedure for Appropriation Accounts Available for Definite Periods states the following, On September 30$^{th}$ of the 5$^{th}$ fiscal year after the period of availability for obligation of a fixed appropriation account ends, the account shall be closed and any remaining balances (whether obligated or unobligated) in the account shall be canceled and thereafter shall not be available for obligation or expenditure for any purpose. An example is provided below:

Fiscal Year (FY) 01 funds will expire September 30, 2021. All FY 01 funds should be drawn down and reported to Payment Management Services (PMS) prior to September 30, 2021. After this date, corrections or cash requests will not be permitted.

## REPORTING REQUIREMENTS

**Annual Federal Financial Report (FFR, SF-425):** The Annual Federal Financial Report (FFR) SF-425 is required and must be submitted through eRA Commons no later than 90 days after the end of the calendar quarter in which the budget period ends. The FFR for this budget period is due to the GMS/GMO by **September 30, 2017**. Reporting timeframe is **June 1, 2016** through **May 31, 2017**.

The FFR should only include those funds authorized and disbursed during the timeframe covered by the report. The final FFR must indicate the exact balance of unobligated funds and may not reflect any unliquidated obligations. There must be no discrepancies between the final FFR expenditure data and the Payment Management System's (PMS) cash transaction data. All Federal reporting in PMS is unchanged.

Failure to submit the required information in a timely manner may adversely affect the future funding of this project. If the information cannot be provided by the due date, the grantee is required to contact the Grants Officer listed in the contacts section of this notice before the due date.

FFR (SF-425) instructions for CDC Grantees are available at http://grants.nih.gov/grants/forms.htm. For further information, contact GrantsInfo@nih.gov. Additional resources concerning the eFSR/FFR system, including a User Guide and an on-line demonstration, can be found on the eRA Commons Support Page: http://grants.nih.gov/support/.

**Performance Reporting:** The HHS Public Health Services Non-Competing Continuation Progress Report (PHS2590) is due no later than (NLT) 120 days prior to the end of the budget period or the date January 31, 2017, identified in the solicitation guidance. This report also serves as the continuation application. This report should include the information specified in the FOA. All reports (original and two copies) must be submitted to the CDC Grants Management Specialist identified below under Staff Contacts. The following hyperlink provides the necessary forms for completion: http://grants1.nih.gov/grants/forms.htm. Grantees must also submit a final performance report for closeout purposes.

**Audit Requirement:**
Domestic Organizations ***(including US-based organizations implementing projects with foreign components)***: An organization that expends $750,000 or more in a fiscal year in Federal awards shall have a single or program-specific audit conducted for that year in accordance with the provisions of 45 CFR Part 75. The audit period is an organization's fiscal year. The audit must be completed along with a data collection form (SF-SAC), and the reporting package shall be submitted within the earlier of 30 days after receipt of the auditor's report(s), or nine (9) months after the end of the audit period. The audit report must be sent to:

Federal Audit Clearing House Internet Data Entry System
Electronic Submission:
https://harvester.census.gov/facides/(S(0vkw1zaelyzjibnahocga5i0))/account/login.aspx

AND

Procurement & Grants Office, Risk Management & Compliance Activity
Electronic Copy to: OGS.Audit.Resolution@cdc.gov

Audit requirements for Subrecipients to whom 45 CFR 75 Subpart F applies: The grantee must ensure that the subrecipients receiving CDC funds also meet these requirements. The grantee must also ensure to take appropriate corrective action within six months after receipt of the subrecipient audit report in instances of non-compliance with applicable Federal law and regulations (45 CFR 75 Subpart F and HHS Grants Policy Statement). The grantee may consider whether subrecipient audits necessitate adjustment of the grantee's own accounting records. If a subrecipient is not required to have a program-specific audit, the grantee is still required to

perform adequate monitoring of subrecipient activities. The grantee shall require each subrecipient to permit the independent auditor access to the subrecipient's records and financial statements. The grantee must include this requirement in all subrecipient contracts.

Note: The standards set forth in 45 CFR Part 75 Subpart F will apply to audits of fiscal years beginning on or after December 26, 2014.

**Federal Funding Accountability and Transparency Act (FFATA):** In accordance with 2 CFR Chapter 1, Part 170 Reporting Sub-Award And Executive Compensation Information, Prime Awardees awarded a federal grant are required to file a FFATA sub-award report by the end of the month following the month in which the prime awardee awards any sub-grant equal to or greater than $25,000.

Pursuant to 45 CFR Part 75, §75.502, a grant sub-award includes the provision of any commodities (food and non-food) to the sub-recipient where the sub-recipient is required to abide by terms and conditions regarding the use or future administration of those goods. If the sub-awardee merely consumes or utilizes the goods, the commodities are not in and of themselves considered sub-awards.

2 CFR Part 170: http://www.ecfr.gov/cgi-bin/text-idx?tpl=/ecfrbrowse/Title02/2cfr170_main_02.tpl

FFATA: www.fsrs.gov.

Reporting of First-Tier Sub-awards

Applicability: Unless you are exempt (gross income from all sources reported in last tax return is under $300,000), you must report each action that obligates $25,000 or more in Federal funds that does not include Recovery funds (as defined in section 1512(a)(2) of the American Recovery and Reinvestment Act of 2009, Pub. L. 111-5) for a sub-award to an entity. Reporting: Report each obligating action of this award term to www.fsrs.gov. For sub-award information, report no later than the end of the month following the month in which the obligation was made. (For example, if the obligation was made on November 7, 2010, the obligation must be reported by no later than December 31, 2010). You must report the information about each obligating action that the submission instructions posted at www.fsrs.gov specify.

Total Compensation of Recipient Executives: You must report total compensation for each of your five most highly compensated executives for the preceding completed fiscal year, if:

- The total Federal funding authorized to date under this award is $25,000 or more;
- In the preceding fiscal year, you received—
  - 80 percent or more of your annual gross revenues from Federal procurement contracts (and subcontracts) and Federal financial assistance subject to the Transparency Act, as defined at 2 CFR Part 170.320 (and sub-awards); and
  - $25,000,000 or more in annual gross revenues from Federal procurement contracts (and subcontracts) and Federal financial assistance subject to the Transparency Act, as defined at 2 CFR Part 170.320 (and sub-awards); and
  - The public does not have access to information about the compensation of the executives through periodic reports filed under section 13(a) or 15(d) of the Securities Exchange Act of 1934 (15 U.S.C. Part 78m(a), 78o(d)) or section 6104 of the Internal Revenue Code of 1986. (To determine if the public has access to the compensation information, see the U.S. Security and Exchange Commission total compensation filings at http://www.sec.gov/answers/execomp.htm?explorer.event=true).

Report executive total compensation as part of your registration profile at http://www.sam.gov. Reports should be made at the end of the month following the month in which this award is made and annually thereafter.

Total Compensation of Sub-recipient Executives: Unless you are exempt (gross income from all sources reported in last tax return is under $300,000), for each first-tier sub-recipient under this award, you must report the names and total compensation of each of the sub-recipient’s

five most highly compensated executives for the sub-recipient's preceding completed fiscal year, if:

- In the sub-recipient's preceding fiscal year, the sub-recipient received—
  - 80 percent or more of its annual gross revenues from Federal procurement contracts (and subcontracts) and Federal financial assistance subject to the Transparency Act, as defined at 2 CFR Part 170.320 (and sub-awards); and
  - $25,000,000 or more in annual gross revenues from Federal procurement contracts (and subcontracts), and Federal financial assistance subject to the Transparency Act (and sub-awards); and
  - The public does not have access to information about the compensation of the executives through periodic reports filed under section 13(a) or 15(d) of the Securities Exchange Act of 1934 (15 U.S.C. Part 78m(a), 78o(d)) or section 6104 of the Internal Revenue Code of 1986. (To determine if the public has access to the compensation information, see the U.S. Security and Exchange Commission total compensation filings at http://www.sec.gov/answers/execomp.htm).

You must report sub-recipient executive total compensation to the grantee by the end of the month following the month during which you make the sub-award. For example, if a sub-award is obligated on any date during the month of October of a given year (i.e., between October 1st and 31st), you must report any required compensation information of the sub-recipient by November 30th of that year.

Definitions:

- Entity means all of the following, as defined in 2 CFR Part 25 (Appendix A, Paragraph(C)(3)):
  - Governmental organization, which is a State, local government, or Indian tribe;
  - Foreign public entity;
  - Domestic or foreign non-profit organization;
  - Domestic or foreign for-profit organization;
  - Federal agency, but only as a sub-recipient under an award or sub-award to a non-Federal entity.

- Executive means officers, managing partners, or any other employees in management positions.

- Sub-award: a legal instrument to provide support to an eligible sub-recipient for the performance of any portion of the substantive project or program for which the grantee received this award. The term does not include the grantees procurement of property and services needed to carry out the project or program (for further explanation, see 45 CFR Part 75). A sub-award may be provided through any legal agreement, including an agreement that the grantee or a sub-recipient considers a contract.

- Sub-recipient means an entity that receives a sub-award from you (the grantee) under this award; and is accountable to the grantee for the use of the Federal funds provided by the sub-award.

- Total compensation means the cash and non-cash dollar value earned by the executive during the grantee's or sub-recipient's preceding fiscal year and includes the following (for more information see 17 CFR Part 229.402(c)(2)):
  - Salary and bonus
  - Awards of stock, stock options, and stock appreciation rights. Use the dollar amount recognized for financial statement reporting purposes with respect to the fiscal year in accordance with the Statement of Financial Accounting Standards No. 123 (Revised 2004) (FAS 123R), Shared Based Payments.
  - Earnings for services under non-equity incentive plans. This does not include group life, health, hospitalization or medical reimbursement plans

that do not discriminate in favor of executives, and are available generally to all salaried employees.
  - Change in pension value. This is the change in present value of defined benefit and actuarial pension plans.
  - Above-market earnings on deferred compensation which is not tax-qualified.
  - Other compensation, if the aggregate value of all such other compensation (e.g. severance, termination payments, value of life insurance paid on behalf of the employee, perquisites or property) for the executive exceeds $10,000.

## GENERAL REQUIREMENTS

**Travel Cost**: In accordance with HHS Grants Policy Statement, travel costs are only allowable where such travel will provide direct benefit to the project or program. There must be a direct benefit imparted on behalf of the traveler as it applies to the approved activities of the NoA. To prevent disallowance of cost, the grantee is responsible for ensuring that only allowable travel reimbursements are applied in accordance with their organization's established travel policies and procedures. Grantees approved policies must meet the requirements of 45 CFR Part 75, as applicable.

**Food and Meals**: Costs associated with food or meals are allowable when consistent with applicable federal regulations and HHS policies and guidance, which can be found at http://www.hhs.gov/asfr/ogapa/acquisition/effspendpol_memo.html. In addition, costs must be proposed in accordance with grantee approved policies and a determination of reasonableness has been performed by the grantees. Grantee approved policies must meet the requirements of 45 CFR Part 75, as applicable.
**Prior Approval**: All requests, which require prior approval, must bear the signature of an authorized official of the business office of the grantee organization. The grantee must submit these requests by or no later than 120 days prior to this budget period's end date. Additionally, any requests involving funding issues must include an itemized budget and a narrative justification of the request.

The following types of requests require prior approval.
- Use of unobligated funds from prior budget period (Carryover)
- Lift funding restriction, withholding, or disallowance
- Redirection of funds
- Change in scope
- Implement a new activity or enter into a sub-award that is not specified in the approved budget
- Apply for supplemental funds
- Change in key personnel
- Extensions
- Conferences or meetings that were not specified in the approved budget

Templates for prior approval requests can be found at:
http://www.cdc.gov/grants/alreadyhavegrant/priorapprovalrequests.html

**Key Personnel**: In accordance with 45 CFR Part 75.308, CDC grantees must obtain prior approval from CDC for (1) change in the project director/principal investigator, business official, authorized organizational representative or other key persons specified in the FOA, application or award document; and (2) the disengagement from the project for more than three months, or a 25 percent reduction in time devoted to the project, by the approved project director or principal investigator.
**Inventions**: Acceptance of grant funds obligates grantees to comply with the standard patent rights clause in 37 CFR Part 401.14.

**Publications**: Publications, journal articles, etc. produced under a CDC grant support project must bear an acknowledgment and disclaimer, as appropriate, for example:

This publication (journal article, etc.) was supported by the Grant or Cooperative

Agreement Number, **DP006255**, funded by the Centers for Disease Control and Prevention. Its contents are solely the responsibility of the authors and do not necessarily represent the official views of the Centers for Disease Control and Prevention or the Department of Health and Human Services.

**Acknowledgment Of Federal Support**: When issuing statements, press releases, requests for proposals, bid solicitations and other documents describing projects or programs funded in whole or in part with Federal money, all awardees receiving Federal funds, including and not limited to State and local governments and grantees of Federal research grants, shall clearly state:

- percentage of the total costs of the program or project which will be financed with Federal money
- dollar amount of Federal funds for the project or program, and
- percentage and dollar amount of the total costs of the project or program that will be financed by non-governmental sources.

**Copyright Interests Provision:** This provision is intended to ensure that the public has access to the results and accomplishments of public health activities funded by CDC. Pursuant to applicable grant regulations and CDC's Public Access Policy, Recipient agrees to submit into the National Institutes of Health (NIH) Manuscript Submission (NIHMS) system an electronic version of the final, peer-reviewed manuscript of any such work developed under this award upon acceptance for publication, to be made publicly available no later than 12 months after the official date of publication. Also at the time of submission, Recipient and/or the Recipient's submitting author must specify the date the final manuscript will be publicly accessible through PubMed Central (PMC). Recipient and/or Recipient's submitting author must also post the manuscript through PMC within twelve (12) months of the publisher's official date of final publication; however the author is strongly encouraged to make the subject manuscript available as soon as possible. The recipient must obtain prior approval from the CDC for any exception to this provision.

The author's final, peer-reviewed manuscript is defined as the final version accepted for journal publication, and includes all modifications from the publishing peer review process, and all graphics and supplemental material associated with the article. Recipient and its submitting authors working under this award are responsible for ensuring that any publishing or copyright agreements concerning submitted articles reserve adequate right to fully comply with this provision and the license reserved by CDC. The manuscript will be hosted in both PMC and the CDC Stacks institutional repository system. In progress reports for this award, recipient must identify publications subject to the CDC Public Access Policy by using the applicable NIHMS identification number for up to three (3) months after the publication date and the PubMed Central identification number (PMCID) thereafter.

**Disclaimer for Conference/Meeting/Seminar Materials:** Disclaimers for conferences/meetings, etc. and/or publications: If a conference/meeting/seminar is funded by a grant, cooperative agreement, sub-grant and/or a contract the grantee must include the following statement on conference materials, including promotional materials, agenda, and internet sites:

> Funding for this conference was made possible (in part) by the Centers for Disease Control and Prevention. The views expressed in written conference materials or publications and by speakers and moderators do not necessarily reflect the official policies of the Department of Health and Human Services, nor does the mention of trade names, commercial practices, or organizations imply endorsement by the U.S. Government.

**Logo Use for Conference and Other Materials:** Neither the Department of Health and Human Services (HHS) nor the CDC logo may be displayed if such display would cause confusion as to the funding source or give false appearance of Government endorsement. Use of the HHS name or logo is governed by U.S.C. Part 1320b-10, which prohibits misuse of the HHS name and emblem in written communication. A non-federal entity is unauthorized to use the HHS name or logo governed by U.S.C. Part 1320b-10. The appropriate use of the HHS logo is subject to review and approval of the HHS Office of the Assistant Secretary for Public Affairs (OASPA). Moreover, the HHS Office of the Inspector General has authority to impose civil monetary penalties for violations (42 CFR Part 1003). Accordingly, neither the HHS nor the CDC logo can be used by the grantee without the express, written consent of either the CDC Project Officer or the CDC Grants Management Officer. It is the responsibility of the grantee to request consent for use of the logo in sufficient detail to ensure a complete depiction and disclosure of all uses of the

Government logos. In all cases for utilization of Government logos, the grantee must ensure written consent is received from the Project Officer and/or the Grants Management Officer. Further, the HHS and CDC logos cannot be used by the grantee without a license agreement setting forth the terms and conditions of use.

**Equipment and Products**: To the greatest extent practicable, all equipment and products purchased with CDC funds should be American-made. CDC defines equipment as tangible non-expendable personal property (including exempt property) charged directly to an award having a useful life of more than one year AND an acquisition cost of $5,000 or more per unit. However, consistent with grantee policy, a lower threshold may be established. Please provide the information to the Grants Management Officer to establish a lower equipment threshold to reflect your organization's policy.

The grantee may use its own property management standards and procedures, provided it observes provisions of in applicable grant regulations found at 45 CFR Part 75.

**Federal Information Security Management Act (FISMA):** All information systems, electronic or hard copy, that contain federal data must be protected from unauthorized access. This standard also applies to information associated with CDC grants. Congress and the OMB have instituted laws, policies and directives that govern the creation and implementation of federal information security practices that pertain specifically to grants and contracts. The current regulations are pursuant to the Federal Information Security Management Act (FISMA), Title III of the E-Government Act of 2002, PL 107-347.

FISMA applies to CDC grantees only when grantees collect, store, process, transmit or use information on behalf of HHS or any of its component organizations. In all other cases, FISMA is not applicable to recipients of grants, including cooperative agreements. Under FISMA, the grantee retains the original data and intellectual property, and is responsible for the security of these data, subject to all applicable laws protecting security, privacy, and research. If/When information collected by a grantee is provided to HHS, responsibility for the protection of the HHS copy of the information is transferred to HHS and it becomes the agency's responsibility to protect that information and any derivative copies as required by FISMA. For the full text of the requirements under Federal Information Security Management Act (FISMA), Title III of the E-Government Act of 2002 Pub. L. No. 107-347, please review the following website: http://frwebgate.access.gpo.gov/cgi-bin/getdoc.cgi?dbname=107_cong_public_laws&docid=f:publ347.107.pdf

**Pilot Program for Enhancement of Contractor Employee Whistleblower Protections:**
Grantees are hereby given notice that the 48 CFR section 3.908, implementing section 828, entitled "Pilot Program for Enhancement of Contractor Employee Whistleblower Protections," of the National Defense Authorization Act (NDAA) for Fiscal Year (FY) 2013 (Pub. L. 112-239, enacted January 2, 2013), applies to this award.

Federal Acquisition Regulations
As promulgated in the Federal Register, the relevant portions of 48 CFR section 3.908 read as follows (note that use of the term "contract," "contractor," "subcontract," or "subcontractor" for the purpose of this term and condition, should be read as "grant," "grantee," "subgrant," or "subgrantee"):

3.908 Pilot program for enhancement of contractor employee whistleblower protections.

3.908-1 Scope of section.
(a) This section implements 41 U.S.C. 4712.

(b) This section does not apply to-
- (1) DoD, NASA, and the Coast Guard; or
- (2) Any element of the intelligence community, as defined in section 3(4) of the National Security Act of 1947 (50 U.S.C. 3003(4)). This section does not apply to any disclosure made by an employee of a contractor or subcontractor of an element of the intelligence community if such disclosure-
    - (i) Relates to an activity of an element of the intelligence community; or
    - (ii) Was discovered during contract or subcontract services provided to an element of the intelligence community.

3.908-2 Definitions.
As used in this section-
"Abuse of authority" means an arbitrary and capricious exercise of authority that is inconsistent with the mission of the executive agency concerned or the successful performance of a contract of such agency.

"Inspector General" means an Inspector General appointed under the Inspector General Act of 1978 and any Inspector General that receives funding from, or has oversight over contracts awarded for, or on behalf of, the executive agency concerned.

3.908-3 Policy.
(a) Contractors and subcontractors are prohibited from discharging, demoting, or otherwise discriminating against an employee as a reprisal for disclosing, to any of the entities listed at paragraph (b) of this subsection, information that the employee reasonably believes is evidence of gross mismanagement of a Federal contract, a gross waste of Federal funds, an abuse of authority relating to a Federal contract, a substantial and specific danger to public health or safety, or a violation of law, rule, or regulation related to a Federal contract (including the competition for or negotiation of a contract). A reprisal is prohibited even if it is undertaken at the request of an executive branch official, unless the request takes the form of a non-discretionary directive and is within the authority of the executive branch official making the request.

(b) Entities to whom disclosure may be made.

(1) A Member of Congress or a representative of a committee of Congress.
(2) An Inspector General.
(3) The Government Accountability Office.
(4) A Federal employee responsible for contract oversight or management at the relevant agency.
(5) An authorized official of the Department of Justice or other law enforcement agency.
(6) A court or grand jury.
(7) A management official or other employee of the contractor or subcontractor who has the responsibility to investigate, discover, or address misconduct.

(c) An employee who initiates or provides evidence of contractor or subcontractor misconduct in any judicial or administrative proceeding relating to waste, fraud, or abuse on a Federal contract shall be deemed to have made a disclosure.

3.908-9 Contract clause.
Contractor Employee Whistleblower Rights and Requirement to Inform Employees of Whistleblower Rights (Sept. 2013)

(a) This contract and employees working on this contract will be subject to the whistleblower rights and remedies in the pilot program on Contractor employee whistleblower protections established at 41 U.S.C. 4712 by section 828 of the National Defense Authorization Act for Fiscal Year 2013 (Pub. L. 112-239) and FAR 3.908.

(b) The Contractor shall inform its employees in writing, in the predominant language of the workforce, of employee whistleblower rights and protections under 41 U.S.C. 4712, as described in section 3.908 of the Federal Acquisition Regulation.

(c) The Contractor shall insert the substance of this clause, including this paragraph (c), in all subcontracts over the simplified acquisition threshold.

## PAYMENT INFORMATION

**Automatic Drawdown (Direct/Advance Payments):** Payment under this award will be made available through the Department of Health and Human Services (HHS) Payment Management System (PMS). PMS will forward instructions for obtaining payments.

PMS correspondence, mailed through the U.S. Postal Service, should be addressed as follows:

Director, Division of Payment Management
P.O. Box 6021

Rockville, MD 20852
Phone Number: (877) 614-5533
Email: PMSSupport@psc.gov
Website: http://www.dpm.psc.gov/help/help.aspx?explorer.event=true

**Note**: To obtain the contact information of PMS staff within respective Payment Branches refer to the links listed below:

- University and Non-Profit Payment Branch:
  http://www.dpm.psc.gov/contacts/dpm_contact_list/univ_nonprofit.aspx?explorer.event=true

- Governmental and Tribal Payment Branch:
  http://www.dpm.psc.gov/contacts/governmental_and_tribal.aspx?explorer.event=true

- Cross Servicing Payment Branch:
  http://www.dpm.psc.gov/contacts/cross_servicing.aspx?explorer.event=true

- International Payment Branch:
  Bhavin Patel (301) 492-4918
  Email: Bhavin.patel@psc.hhs.gov

If a carrier other than the U.S. Postal Service is used, such as United Parcel Service, Federal Express, or other commercial service, the correspondence should be addressed as follows:

U.S. Department of Health and Human Services
Division of Payment Management
7700 Wisconsin Avenue, Suite 920
Bethesda, MD  20814

To expedite your first payment from this award, attach a copy of the Notice of Grant/Cooperative Agreement to your payment request form.

**Payment Management System Subaccount:** Effective October 1, 2013, a new HHS policy on subaccounts requires the CDC setup payment subaccounts within the Payment Management System (PMS) for all new grant awards. Funds awarded in support of approved activities have been obligated in a newly established subaccount in the PMS, herein identified as the "P Account". A P Account is a subaccount created specifically for the purpose of tracking designated types of funding in the PMS.

All award funds must be tracked and reported separately. Funds must be used in support of approved activities in the FOA and the approved application.
The grant document number and subaccount title (below) must be known in order to draw down funds from this P Account.

Grant Document Number: **16DP006255**
Subaccount Title: **DP16004COOPAGREEFY16**

**Acceptance of the Terms of an Award:** By drawing or otherwise obtaining funds from the grant Payment Management Services, the grantee acknowledges acceptance of the terms and conditions of the award and is obligated to perform in accordance with the requirements of the award. If the recipient cannot accept the terms, the recipient should notify the Grants Management Officer within thirty (30) days of receipt of this award notice.

**Certification Statement**: By drawing down funds, the grantee certifies that proper financial management controls and accounting systems, to include personnel policies and procedures, have been established to adequately administer Federal awards and funds drawn down. Recipients must comply with all terms and conditions outlined in their NoA, including grant policy terms and conditions contained in applicable
HHS Grant Policy Statements, and requirements imposed by program statutes and regulations and HHS grants administration regulations, as applicable; as well as any regulations or limitations in any applicable appropriations acts.

**Roles and Responsibilities:** Grants Management Specialists/Officers (GMO/GMS) and Program/Project Officers (PO) work together to award and manage CDC grants and cooperative agreements. From the pre-planning stage to closeout of an award, grants management and program staff have specific roles and responsibilities for each phase of the grant cycle. The GMS/GMO is responsible for the business management and administrative functions. The PO is responsible for the programmatic, scientific, and/or technical aspects. The purpose of this factsheet is to distinguish between the roles and responsibilities of the GMO/GMS and the PO to provide a description of their respective duties.

**Grants Management Officer:** The GMO is the federal official responsible for the business and other non-programmatic aspects of grant awards including:

- Determining the appropriate award instrument, i.e.; grant or cooperative agreement
- Determining if an application meets the requirements of the FOA
- Ensuring objective reviews are conducted in an above-the-board manner and according to guidelines set forth in grants policy
- Ensuring grantee compliance with applicable laws, regulations, and policies
- Negotiating awards, including budgets
- Responding to grantee inquiries regarding the business and administrative aspects of an award
- Providing grantees with guidance on the closeout process and administering the closeout of grants
- Receiving and processing reports and prior approval requests such as changes in funding, carryover, budget redirection, or changes to the terms and conditions of an award
- Maintaining the official grant file and program book

The GMO is the only official authorized to obligate federal funds and is responsible for signing the NoA, including revisions to the NoA that change the terms and conditions. The GMO serves as the counterpart to the business officer of the recipient organization.

**GMO Contact:** See Staff Contacts below for the assigned GMO

**Grants Management Specialist:** The GMS is the federal staff member responsible for the day-to-day management of grants and cooperative agreements. The GMS is the primary contact of recipients for business and administrative matters pertinent to grant awards. Many of the functions described above are performed by the GMS on behalf of the GMO.

**GMS Contact:** See Staff Contacts below for the assigned GMS

**Program/Project Officer:** The PO is the federal official responsible for the programmatic, scientific, and/or technical aspects of grants and cooperative agreements including:

- The development of programs and FOAs to meet the CDC's mission
- Providing technical assistance to applicants in developing their applications e.g. explanation of programmatic requirements, regulations, evaluation criteria, and guidance to applicants on possible linkages with other resources
- Providing technical assistance to grantees in the performance of their project
- Post-award monitoring of grantee performance such as review of progress reports, review of prior approval requests, conducting site visits, and other activities complementary to those of the GMO/GMS

**Scientific Program Official:** The SPO will be responsible for the normal scientific and programmatic stewardship of grants and cooperative agreements including:

- Being named in this NoA as the Program Official to provide oversight and assure overall scientific and programmatic stewardship of the award;
- Collaborating, as appropriate, with the recipient in all stages of the program, and providing post-award scientific review and administrative guidance;
- Monitoring performance against approved project objectives.
- Making recommendations on requests for changes in scope, objectives, and/or budgets that deviate from the approved peer-reviewed application;

- Assessing the public health impact of the research conducted under this funding opportunity announcement and promoting translation of promising practices, programs, interventions, and other results from the research.
- Serving as the primary point of contact on official award-related activities, including an annual review of the grantee's performance as part of the request for continuation application.

**Programmatic Contacts:**

Suzianne Garner, Project Officer
Centers for Disease Control
Program Services Branch
4770 Buford Hwy, Bldg. 107
Atlanta, GA 30341
Telephone: 770-488-5044
Fax: 770-488-6500
Email: SGarner@cdc.gov

Natalie Darling, Scientific Program Official
Centers for Disease Control
Program Services Branch
4770 Buford Hwy, Bldg. 107
Atlanta, GA 30341
Telephone: 770-488-5740
Fax: 770-488-7863
Email: NDarling@cdc.gov

**Staff Contacts:**

Monique McEwen, Grants Management Specialist
Office of Grants Services (OGS)
Office of Financial Resources (OFR)
Office of the Chief Operating Officer (OCOO)
Centers for Disease Control and Prevention (CDC)
Email: mmcewen@cdc.gov Phone: 770-488-2617

Roslyn Curington, Grants Management Officer
Office of Grants Services (OGS)
Office of Financial Resources (OFR)
Office of the Chief Operating Officer (OCOO)
Centers for Disease Control and Prevention (CDC)
Email: RCurington@cdc.gov Phone: 770-488-2832

**STAFF CONTACTS**

**Grants Management Specialist**: Monique Mcewen
Center for Disease Control and Prevention
Procuerment and Grants Office
Koger Center/Colgate Bldg/Room 2908
MS E18
Atlanta, GA 30331
**Email**: itn8@cdc.gov **Phone**: 770-488-2617 **Fax**: 770-488-2847

**Grants Management Officer:** Roslyn Curington
Centers for Disease Control and Prevention
OD/OCOO/PGO/AABI
Koger Center, Colgate Builder
2920 Brandywine Road, Mailstop E15
Atlanta, GA 30341

**Email:** rcurington@cdc.gov **Phone**: (770) 488-2832 **Fax**: 770-488-2868

**SPREADSHEET SUMMARY**
**GRANT NUMBER:** 1U18DP006255-01

**INSTITUTION:** ARIZONA STATE UNIVERSITY-TEMPE CAMPUS

| Budget | Year 1 | Year 2 |
|---|---|---|
| Salaries and Wages | $458,302 | |
| Fringe Benefits | $119,124 | |
| Personnel Costs (Subtotal) | $577,426 | |
| Supplies | $57,558 | |
| Travel Costs | $27,332 | |
| Other Costs | $211,636 | |
| Consortium/Contractual Cost | $342,164 | |
| TOTAL FEDERAL DC | $1,216,116 | $0 |
| TOTAL FEDERAL F&A | $498,834 | $498,834 |
| TOTAL COST | $1,714,950 | $498,834 |
